# A double-blind, placebo-controlled intervention trial of 3 and 10 mg sublingual melatonin for post-concussion syndrome in youths (PLAYGAME): study protocol for a randomized controlled trial

**DOI:** 10.1186/1745-6215-15-271

**Published:** 2014-07-07

**Authors:** Karen M Barlow, Brian L Brooks, Frank P MacMaster, Adam Kirton, Trevor Seeger, Michael Esser, Susan Crawford, Alberto Nettel-Aguirre, Roger Zemek, Mikrogianakis Angelo, Valerie Kirk, Carolyn A Emery, David Johnson, Michael D Hill, Jeff Buchhalter, Brenda Turley, Lawrence Richer, Robert Platt, Jamie Hutchison, Deborah Dewey

**Affiliations:** 1 Alberta Children’s Hospital Research Institute, University of Calgary, Room 293, Heritage Medical Research Building 3330 Hospital Drive NW, Calgary, AB T2N 4N1, Canada; 2 Pediatric Emergency Medicine, Children’s Hospital of Eastern Ontario, 01 Smyth Road, Ottawa, Ontario K1H 8L1, Canada; 3 Faculty of Kinesiology, 2500 University Drive NW, Calgary, AB T2N 1N4, Canada; 4 Hotchkiss Brain Institute, University of Calgary, Health Research Innovation Centre, Room 1A10, 3330 Hospital Drive NW, Calgary T2N 4N1 Alberta Canada; 5 Department of Pediatric Neurology, University of Alberta, Room 1D1, 8440 112 Street, Edmonton T6G 2B7 Alberta, Canada; 6 Department of Statistics, Montreal Children’s Hospital Research Institute, McGill University, 2300 Tupper Street, Montreal, Quebec H3H 1P3, Canada; 7 Department of Pediatrics, Department of Critical Care Medicine, The Hospital for Sick Children, 555 University Ave. 2nd Floor, Atrium - Room 2830A, Toronto, Ontario M5G 1X8, Canada; 8 Alberta Children’s Hospital, 2888 Shaganappi Trail NW, Calgary, Alberta T3B 6A8, Canada

**Keywords:** Concussion, Traumatic brain injury, Melatonin, Placebo, Pediatric, Randomized controlled trial

## Abstract

**Background:**

By the age of sixteen, one in five children will sustain a mild traumatic brain injury also known as concussion. Our research found that one in seven school children with mild traumatic brain injury suffer post-concussion syndrome symptoms for three months or longer. Post-concussion syndrome is associated with significant disability in the child and his/her family and yet there are no evidence-based medical treatments available. Melatonin has several potential mechanisms of action that could be useful following mild traumatic brain injury, including neuroprotective effects. The aim of this study is to determine if treatment with melatonin improves post-concussion syndrome in youths following mild traumatic brain injury. Our hypothesis is that treatment of post-concussion syndrome following mild traumatic brain injury with 3 or 10 mg of sublingual melatonin for 28 days will result in a decrease in post-concussion syndrome symptoms compared with placebo.

**Methods/Design:**

Ninety-nine youths with mild traumatic brain injury, aged between 13 and 18 years, who are symptomatic at 30 days post-injury will be recruited. This study will be conducted as a randomized, double blind, placebo-controlled superiority trial of melatonin. Three parallel treatment groups will be examined with a 1:1:1 allocation: sublingual melatonin 3 mg, sublingual melatonin 10 mg, and sublingual placebo. Participants will receive treatment for 28 days. The primary outcome is a change on the Post-Concussion Symptom Inventory (Parent and Youth). The secondary outcomes will include neurobehavioral function, health-related quality of life and sleep. Neurophysiological and structural markers of change, using magnetic resonance imaging techniques and transcranial magnetic stimulation, will also be investigated.

**Discussion:**

Melatonin is a safe and well-tolerated agent that has many biological properties that may be useful following a traumatic brain injury. This study will determine whether it is a useful treatment for children with post-concussion syndrome. Recruitment commenced on 4 December 2014.

**Trial registration:**

This trial was registered on 6 June 2013 at ClinicalTrials.gov. Registration number: NCT01874847.

## Background

Traumatic brain injury (TBI) is one of the most common causes of neurological morbidity and it is more common in childhood and adolescence than at any other time of life [1-4]. Mild traumatic brain injury (mTBI) is the acute neurophysiological effect of blunt impact or other mechanical energy applied to the head, such as from sudden acceleration, deceleration or rotational forces [5,6]. It accounts for 90% of all TBIs [7]. Epidemiological studies suggest that one in five children will experience a mTBI by the age of 10, [1,8] and 799 out of 100,000 children under 14 years visit the emergency department (ED) with a mTBI in the United States [2] and Canada [9]. Falls (51%) and sports-related activities (25%) are the commonest causes [2,10,11].

One in seven school children sustaining an mTBI will suffer post-concussion syndrome (PCS) symptoms for three months or longer [10]. PCS is a combination of clinical symptoms including physical (such as headaches), cognitive (such as learning and/or memory dysfunction), and behavioral (such as mood) disturbances [7,10,12]. It is associated with significant disability in the child and his/her family [5,12-17]. It has been shown that 2% of mTBI children continue to have PCS symptoms one year later [10]. Using these figures, we estimate that annually over a 1000 children in Canada have PCS for over a year due to a ‘mild’ TBI and yet there are no evidence-based medical treatments available [18]. This suggests an urgent need to develop novel treatment options to improve outcomes for children suffering from PCS [19]. Furthermore, our neurobiological understanding of PCS is lacking [6,18], and routine clinical tests are not informative and so are not helpful in guiding treatment.

The complex pathophysiology of mTBI is well described [19-26], but the explanations for prolonged PCS are unclear [21,27]. The mechanisms leading to neuronal dysfunction, cell death and altered connectivity include: oxidative stress, metabolic dysfunction, neuroinflammation, axonal damage and alterations in cerebral blood flow [19,21,28]. Most animal studies demonstrate recovery from mTBI within 7 to 15 days [22,29,30], similar to the clinical experience of the majority of humans [6,10,12]. However, the pathophysiological explanations for prolonged PCS, seen in 11% of children with mTBI, have been elusive [10,27]. Recent animal and human research suggest that the explanations for the persistent PCS symptoms may be due to alterations in neuronal circuitry and neurotransmission [29-36].

### Treatment of post-concussion syndrome

There are few evidence-based treatments for PCS and these studies usually do not include children, and so pediatricians have to rely on consensus guidelines for adults [37-44]. Avoidance of repeat injury is the mainstay in any treatment regimen for TBI as is rest until symptoms resolve [18,45]. As PCS symptoms resolve quickly in the majority of people, clinicians do not use pharmacological treatments (except for analgesics) in the first few weeks after injury [38,46-50]. There are few evidence-based treatments for PCS that persists for one month or more [37,40,43,44,51-53], providing the clinician with little guidance for the management of significantly debilitated patients [54]. Treatments are used without conclusive evidence [51] and are chosen to target the most problematic symptom [49,54-60]. A frequently recommended treatment for sleep dysfunction after mTBI is melatonin [61].

### Melatonin as a potential treatment for PCS

Melatonin, naturally produced in the body by the pineal gland, has neuroprotective, analgesic, and anxiolytic properties and is a promising agent in TBI [62-67]. Melatonin’s role in the chronological regulation of major physiological processes (such as the sleep/wake cycle) [68-70] is well accepted. More recently, its therapeutic potential is being explored in other neurobehavioral conditions (for example chronic pain, headaches and anxiety) and TBI [67,71,72]. The many separate biological activities of melatonin are both receptor-mediated (at physiological levels) and non-receptor mediated (especially at supraphysiological levels) [63,72]. It is lipophilic, can cross cell membranes easily [73,74] and its neuroprotective mechanisms include i) reducing oxidative stress (for example decreasing oxidative and nitrosative abuse, lipid peroxidation, and increasing antioxidant enzymes) [75-81], ii) improving mitochondrial function [62,73,74,82,83], iii) inhibiting apoptosis (cell death) [84-86], iv) decreasing the neuroinflammation [64,87,88], and v) decreasing glutamate toxicity via GABA receptors [89-91].

The inherent biochemical and physiological characteristics of the brain, including high polyunsaturated fatty acids and energy requirements, make it particularly susceptible to free radicals mediated insult. Melatonin has been shown to decrease oxidative stress induced by exercise in young athletes [92,93] and in patients with renal failure [94]. It protects against focal and global brain injury in adult and juvenile TBI [87,95-97], ischemic brain injury [98,99], cerebral edema [67,100], spinal cord injury [101,102] and radiation injury [103].

Further, melatonin improves many of the symptoms seen in PCS such as headaches, pain, and anxiety [104] probably via the gamma Aminobuteric acid (GABA)-ergic system and opiate receptors [66,105-107]. It is used to aid sleep in children with disabilities and visual impairment [108]. Melatonin has analgesic properties and has been shown to be useful in adult and pediatric migraine [109,110] and disorders of chronic pain (such as fibromyalgia and irritable bowel syndrome) [111,112]. It is also effective in treating anxiety. In a systematic review, premedication with melatonin significantly decreased preoperative anxiety [113].

The dose of melatonin in clinical pediatric practice ranges between 1 and 10 mg. Receptor-mediated effects occur at physiological doses (for example in children with chronic insomnia effects are achieved at 0.05 to 0.15 mg/kg) [114]. Lower doses do not achieve the same analgesic and anxiolytic effects [109,111,115-118]. In order to saturate melatonin receptors and achieve non-receptor mediated effects, supra-physiological doses are required [114,119,120]. A dose of 10 mg melatonin is likely to achieve this and yet stay within clinically-accepted parameters [70].

### Pilot data using melatonin in PCS

We found that children with prolonged PCS and headaches had a significant response to melatonin treatment [61,121]. Post-traumatic headaches (PTH) are thought to be particularly resistant to treatment [55,122,123]. Few studies have specifically analyzed how patients respond to treatment [124,125], and none of these were in children. Our study aimed to: 1) describe the headache characteristics of PCS in children and 2) their response to pharmacological treatments [61]. A retrospective chart review of 48 children treated for PTH since 2007 was performed. The mean age was 14.1 years (SD 3.1) and 66% were female. The time since injury was 10.6 (SD 8.1) months. Melatonin was used as a first-line treatment where sleep dysfunction was a comorbidity. A total of 15 out of 18 children responded to melatonin treatment. Seven children were treated with 3 to 5 mg of sublingual melatonin; 11 children were treated with 6 to 10 mg. Significantly more children responded to treatment with melatonin (83%) when compared with the other treatments used (*P* <0.05) and no serious side effects were reported. As these children were on average 10.2 months post-injury, it is very unlikely that this response was due to time alone.

In summary, melatonin has potential as a safe therapeutic candidate for the treatment of PCS in children. It has efficacy in many of symptoms commonly encountered in PCS. In preliminary work, we found that children with prolonged PCS and headaches had a significant response to sublingual melatonin treatment [64]. Melatonin’s therapeutic potentials in mTBI include: 1) as a free radical scavenger and broad-spectrum antioxidant [75,76,82,88,126] and 2) symptomatically via the GABAergic system and opiate receptors [66,95,105,106,127]. The aim of this trial is to determine if treatment with melatonin improves PCS following mTBI in youths.

## Methods/Design

We hypothesize that the treatment of children with PCS following mTBI with 3 or 10 mg of melatonin for 28 days will result in a decrease in PCS symptoms as compared with a placebo. The primary research question will be: Does the treatment of children with PCS symptoms following mTBI with 3 or 10 mg of sublingual melatonin for 28 days result in a decrease in PCS (physical, cognitive and behavioral) symptoms as compared with a placebo? The secondary research questions will be: 1) Is there a dose-response relationship? and 2) Is the treatment effect independent of the effect on sleep?

### Basic study design

**Figure 1:**
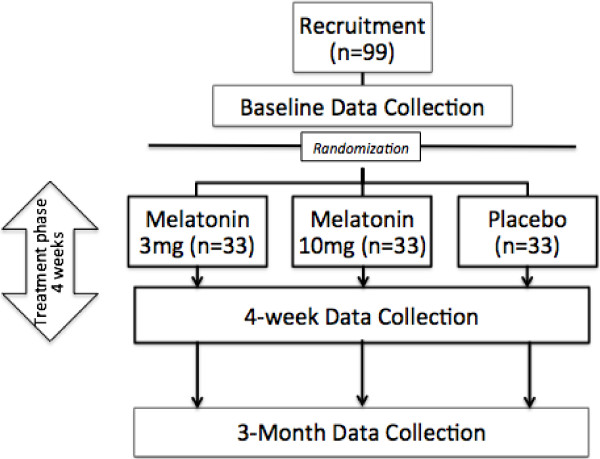
**Study flow diagram.** The study will recruit 99 participants from Alberta Children’s Hospital Emergency Department and surrounding sports medicine and pediatric centers. Participants are randomized after baseline data collection to melatonin 3 mg, melatonin 10 mg, or placebo in a 1:1:1 ratio. There is a four-week treatment phase. Baseline assessments (day 30 +/-10 days) include standard history and clinical assessment, neurocognitive assessment, CHQ, BASC-2, BRIEF and actigraphy. During treatment the participant will keep daily sleep and treatment logs. Actigraphy will continue through treatment. Weekly telephone calls will be made to monitor adverse effects. Post-treatment assessments occur during days 59 to 70 and a final telephone follow-up will occur at day 90 (+/-7 days). CHQ (Child Health Questionnaire), BASC-2 (Behavioral assessment system for children), BRIEF (Behavior Rating Inventory of Executive Function).

This study will be conducted as a randomized, double blind, placebo-controlled superiority trial. Three parallel treatment groups will be examined with a 1:1:1 allocation: 1) sublingual placebo, 2) sublingual melatonin 3 mg, and 3) sublingual melatonin 10 mg, see Figure 1. Individuals will be allocated to treatment groups using a randomization sequence that will be created in variable random block sizes (multiples of 3: 3, 6 and 9) to aid in the concealment of the next allocation, using random number-generating software. Participants, parents and investigators will be blinded to treatment groups. The primary endpoint is the change on the Post-Concussion Symptom Inventory Score for the parent (PCSI-P) and youth (PCSI-Y). Secondary outcome measures are listed in Table 1. The design allows for dose-dependent response assessment. This study was approved by Health Canada (clinical trial application number: 16391). Ethical permission was granted by the University of Calgary Health Research Ethics Board (number: 13-0372); protocol amendment version 02: 24 March 2014. Trial metadata are given in Addition file 1.

**Table 1:** Secondary outcome measures for the PLAYGAME trial Child Health Questionnaire (CHQ)

**Secondary outcome measures**	
Child Health questionnaire (CHQ): Parent (CHQ-PF50) and Child (CHQ-CH87) [128-131]	Neurocognitive ability (using CNS-Vital Signs [132])
Behavior Assessment System-2 [133,134]	Actigraphy: sleep duration, bed time, sleep time, wake time; longest and shortest sleep time [135-137]
The Behavior Rating Inventory of Executive Function [138]	Adverse outcomes and/or side effects

The study will take place in two academic children’s hospitals in Canada, Alberta Children’s Hospital and Children’s Hospital of Eastern Ontario. The target population will be all children aged between 13 and 18 years with an mTBI who remain symptomatic at 30 days post-injury.

### Subjects

#### Eligibility criteria

Parents and adolescents must provide written informed consent before any study procedures occur. Inclusion and exclusion criteria are shown in Table 2.

**Table 2:** Inclusion and exclusion criteria

**Inclusion criteria**	**Exclusion criteria**
Age 13-18 years inclusive	Previous significant medical history
Mild traumatic brain injury [139]	Previous concussion within 12 months
Symptomatic (increase in PCS symptoms compared with pre-injury) at 30 days post injury	Lactose intolerance, as the placebo contains lactose
	Use of drugs that are likely to affect TMS, fMRI and/or sleep
	Inability to complete questionnaires or evaluation
	Claustrophobia or inability to tolerate MRI
	Contraindications to TMS (including history of seizures, unexplained loss of consciousness, metal in the head and/or implanted brain medical devices, cardiac pacemaker, and so on)

Post Concussion Syndrome (PCS), Transcranial Magnetic Stimulation (TMS), Magnetic Resonance Imaging (MRI), functional MRI (fMRI).

### Interventions

Eligible patients will be randomized in equal proportions between three groups: placebo, 3 mg melatonin and 10 mg melatonin. Medication is taken sublingually at night, one hour before sleep time, for 28 days and will be continued even if there is symptom resolution. The placebo, 3 mg melatonin and 10 mg melatonin sublingual tablets are identically sized white tablets which are peppermint flavored.

### Adherence

Administration of the study pill will occur at home under the supervision of the parent. When the study pill is dispensed, the research coordinator will review the importance of following study guidelines and instructions about taking study pills including timing, storage, and what to do in the event of a missed dose. Methodologies to maximize follow-up and compliance include convenient follow-up times, participant engagement strategies (such as newsletters and a website) and experienced research personnel. Adherence assessments will include a review of the medication log, a pill count every week, and a review of reasons for non-compliance. Unused will be counted and recorded on the appropriate case report form. With regards to concomitant care, there are no restrictions on the use of other medications. All participants will be advised to try to avoid analgesia overuse. Participants will be asked to complete a diary of any medications, medical appointments and alternative therapies.

### Primary outcome measure

The Post-Concussion Symptom Inventory - Parent and Youth (PCSI-P and PCSI-Y). These standardized questionnaires examining 26 symptoms, provide an overall rating of PCS. They have four specific domains: physical, cognitive, emotional and fatigue, and a high level of internal consistency reliability (alpha = 0.92) [140,141]. A change in PCSI scores allows us to account for baseline variability and gender (timeline: pretreatment, mid-treatment (day 14 to 15) and post-treatment (day 30 to 35) and 90 to 120 days post-injury) [142].

Secondary outcome measures (Table 1) will be collected at baseline and at the end of treatment (day 30 to 35). These will include parent and child rating of functional impairment using the 50-item Child Health Questionnaire (CHQ) [128-131], the Behaviour Assessment System for Children – 2 which is a standardized parent report measure of child behavior, and the Behaviour Rating Inventory of Executive Function (BRIEF) to assess daily executive abilities. Neurocognitive ability (including attention, executive functioning, memory, reaction time and information processing speed will be measured using a computerized test, CNS-Vital Signs [132]. Actigraphy will be used to measure: sleep duration, bed time, sleep time, wake time; longest and shortest sleep time [135-137].

### Participant timeline and process

Screening takes place by telephone around 30 days post-injury. Parental consent and youth assent will be obtained by the research assistant in the clinic.

### Sample size

The main objective and hypothesis is based on the difference between the placebo and the 3 mg melatonin groups. The outcome of interest is the change in the PCSI score (day 30 minus day 59) and calls for a test of means between groups using one way analysis of variance (ANOVA). We used data obtained in our epidemiological study to calculate a reliable change score using the Jacobson Truax method [10,143]. A 10-point change on the PCSI-P score indicates a reliable change for subjects who are symptomatic at one month (SD 14.7). Further, we find that in practice a 10-point change is also clinically significant. Using a significance level of alpha = 0.05, expecting a power of 80%, assuming a within group SD of 14.7 and using as an effect size a difference between groups of 10 (the reliable change score), a sample size of 30 per group is required. Allowing for a 10% attrition rate, 33 participants per group are required. Recruitment will occur over three years with 36 to 48 patients being recruited each year.

### Statistical analysis

Baseline demographic and clinical variables will be examined for group differences using ANOVA for continuous variables and chi-square tests for categorical variables. Subsequent analyses will involve controlling for possible confounding factors. All analyses will be done on an intention-to-treat basis (last observation carried forward). Group differences in the change in PCSI scores will be analyzed using ANOVA, with placebo, 3 mg melatonin and 10 mg melatonin as groups. Estimates and corresponding 95% confidence intervals for the *a priori* set pairwise comparisons of interest (placebo versus 3 mg, 3 mg versus 10 mg and/or placebo versus 10 mg) will be provided. Time to symptom resolution (as defined by a PCSI score equal or less than pre-injury*)* will be examined using the Cox proportional hazards model. Adverse events will be tabulated. Complete documentation will be kept on ‘non-completers’, including their reasons for non-completion. Differences between randomized groups for early termination will be descriptively reported. A secondary efficacy analysis will be done on ‘completers’ per protocol only, excluding protocol violations.

Analyses of secondary outcomes will examine group differences with a series of ANOVAs examining changes in scores for: 1) CHQ, 2) BASC-2 (parent), 3) sleep parameters, and 4) cognition (measured by CNS Vital Signs battery: attention, executive functioning, and reaction time, processing speed). Hierarchical multiple-regression modeling will be used to predict symptom improvement by entering sleep parameters (step 1), and treatment group (step 2) in order to determine how predictive treatment is above and beyond any effects of melatonin on sleep. Subgroup analysis will be based on the following dichotomies: personal or family history of migraine, loss of consciousness, and previous history of concussion. Bonferroni correction will be used for *post hoc* analysis. All analyses will be performed using **Statistical Package for the Social Sciences (**SPSS) version 22.0 (SPSS Inc., Chicago, Illinois, United States). Outcome analysis will be performed after all participants have been recruited and reviewed by an independent biostatistician as part of the Data Safety Monitoring Board (DSMB). An interim safety analysis will be performed under the direction of the DSMB.

### Safety and potential risks

Melatonin is available as an over-the-counter sleep aid in Canada. It is well tolerated by humans even at supra-physiological doses [104]. Systematic reviews of melatonin in sleep disorders have found melatonin to be safe in adults and children [144,145]. For all adverse events, there was no significant difference between melatonin and the placebo [144]. Any adverse events in PLAYGAME will be immediately reported to the principal investigator who will report any serious unexpected adverse event to the DSMB.

### Trial management

The Trial Steering Committee (TSC) in accordance with Good Clinical Practice Guidelines will manage the trial (Chair: KMB). All lead investigators and authors of this paper will be steering committee members.

The Trial Management Committee (KMB, BLB, AK, FPM and BT) will be responsible for the day-to-day running of the trial.

The DSMB will be responsible for safeguarding the interests of trial participants, potential participants and investigators (JH, LR and RP).

### Research team responsibilities

KMB (Director of Brain Injury and Rehabilitation Program) is a pediatric neurologist and expert in TBI. She will be responsible for the day-to-day management, adherence to protocol, patient clinical concerns, data interpretation, manuscript preparation and distribution/utilization of trial results. DD is an experienced research team leader and neurobehavioral scientist who, together with MDH, will ensure smooth trial operation, and DJ is an expert in knowledge translation. This research team brings together the expertise of a TBI neuropsychologist BLB (cognitive testing), pediatric emergency medicine MA (ED personnel management, data collection and recruitment), an expert in pediatric sleep disorders and VK (antigraph and sleep logs). Research methodological and biostatistical experience will be provided by SC and ANA (study design, data management and data analysis).

### Expected results

It is expected that children treated with melatonin will have lower scores on the PCSI when compared with the placebo, and will have less behavioral (measured using the Behavioral Assessment System for Children) and functional impairment (measured using the Child Health Questionnaire). We expect to observe a dose-response relationship, with 10 mg melatonin treatment group having significantly lower PCSI scores, faster reaction times and increased processing speed compared with the 3 mg melatonin group. Sleep parameters are not expected to differ between the melatonin groups. It is expected that melatonin will be well tolerated without serious adverse side effects.

## Discussion

Melatonin is available as an over-the-counter sleep aid in Canada. It is well tolerated even at supra-physiological doses. It has many biological properties that make it a promising treatment in traumatic brain injury. This study is a first step in elucidating whether sublingual melatonin is a useful treatment for children with post-concussion syndrome. This study will provide valuable information about the neurobiology of post-concussion syndrome in children, including the neurophysiological and structural properties of the brain during recovery from mTBI (not discussed here).

## Trial status

The trial commenced on 4 December 2013 and is in recruitment. It will run until approximately November 2019.

## Abbreviations

ACH: Alberta Children’s Hospital; ANOVA: Analysis of variance; BASC-2: Behavioral assessment system for children; CHEO: Children’s Hospital of Eastern Ontario; CHQ: Child health questionnaire; CHREB: University of Calgary Health Research Ethics Board; DSMB: Data safety monitoring board; ED: Emergency department; mTBI: Mild traumatic brain injury; PCS: Post-concussion syndrome; PCSI: Post-concussion symptom inventory; PCSI-P: Post-concussion symptom inventory-parent; PCSI-Y: Post-concussion symptom inventory-youth; PTH: Post-traumatic headache; TBI: Traumatic brain injury; TSC: Trial steering committee.

## Competing interests

The authors declare that they have no competing interests.

## Authors’ contributions

KMB: principal investigator, conception and initiation, running of the trial, writing, critical revision, and final approval of manuscript. DD: conception and design of study. DJ: aided in design of study, emergency medicine, and knowledge translation. AK: conception and design of study, transcranial magnetic stimulation. FPM: conception and design of study, functional neuroimaging. BLB: conception and design of study, neuropsychological testing. AM: design of study, recruitment, emergency medicine. VK: conception and design of study, sleep medicine. MH: conception and design of study, trial operations. ME: conception and design of study, trial operations. BT: Implementation, data collection and trial operations. TS: Implementation, transcranial magnetic stimulation data collection and analyses. RZ: Implementation, data collection. SC: Design and analyses of data. MDH: Design and analyses of data, trial operations. ANA: Design and analyses of data. CAE: Independent advisor to trial. JB: Data management, therapeutics. LR: Data safety monitoring. RP: Data safety monitoring. JH: Data safety monitoring. All authors read and approved the final manuscript.

## Supplementary Material

Additional file 1 PLAYGAME trial metadata. Click here for file

## References

[B1] McKinlay A Grace RC Horwood LJ Fergusson DM Ridder EM MacFarlane MR Prevalence of traumatic brain injury among children, adolescents and young adults: prospective evidence from a birth cohort Brain Inj 2008 22 175 181 18240046 10.1080/02699050801888824

[B2] Langlois JA Rutland-Brown W Thomas KE The incidence of traumatic brain injury among children in the United States: differences by race J Head Trauma Rehabil 2005 20 229 15908823 10.1097/00001199-200505000-00006

[B3] Thornhill S Teasdale GM Murray GD McEwen J Roy CW Penny KI Disability in young people and adults one year after head injury: prospective cohort study BMJ 2000 320 1631 1635 10856063 10.1136/bmj.320.7250.1631 PMC27407

[B4] Maas AIR Stocchetti N Bullock R Moderate and severe traumatic brain injury in adults Lancet Neurol 2008 7 728 741 18635021 10.1016/S1474-4422(08)70164-9

[B5] Gagnon I Swaine B Friedman D Forget R Exploring children’s self-efficacy related to physical activity performance after a mild traumatic brain injury J Head Trauma Rehabil 2005 20 436 449 16170252 10.1097/00001199-200509000-00005

[B6] Barkhoudarian G Hovda DA Giza CC The molecular pathophysiology of concussive brain injury Clin Sports Med 2011 30 33 iii 21074080 10.1016/j.csm.2010.09.001

[B7] Heads Up Facts for Physicians about Mild Traumatic Brain Injury http://www.cdc.gov/concussion/headsup/pdf/Facts_for_Physicians_booklet-a.pdf

[B8] Corrigan JD Selassie AW Orman JAL The epidemiology of traumatic brain injury J Head Trauma Rehabil 2010 25 72 20234226 10.1097/HTR.0b013e3181ccc8b4

[B9] Ryu WHA Feinstein A Colantonio A Streiner DL Dawson DR Early identification and incidence of mild TBI in Ontario Can J Neurol Sci 2009 36 429 435 19650352 10.1017/s0317167100007745

[B10] Barlow KM Crawford S Stevenson A Sandhu SS Belanger F Dewey D Epidemiology of postconcussion syndrome in pediatric mild traumatic brain injury Pediatrics 2010 126 e374 e381 20660554 10.1542/peds.2009-0925

[B11] Langlois JA Rutland-Brown W Wald MM The epidemiology and impact of traumatic brain injury: a brief overview J Head Trauma Rehabil 2006 21 375 16983222 10.1097/00001199-200609000-00001

[B12] Yeates KO Taylor HG Rusin J Bangert B Dietrich A Nuss K Wright M Nagin DS Jones BL Longitudinal trajectories of postconcussive symptoms in children with mild traumatic brain injuries and their relationship to acute clinical status Pediatrics 2009 123 735 743 19254996 10.1542/peds.2008-1056 PMC2839361

[B13] Moran LM Taylor HG Rusin J Bangert B Dietrich A Nuss KE Wright M Yeates KO Do postconcussive symptoms discriminate injury severity in pediatric mild traumatic brain injury? J Head Trauma Rehabil 2011 26 348 354 21900857 10.1097/HTR.0b013e3181f8d32e PMC3221328

[B14] Emanuelson I Andersson HE Bjorklund R Stalhammar D Quality of life and post-concussion symptoms in adults after mild traumatic brain injury: a population-based study in western Sweden Acta Neurol Scand 2003 108 332 338 14616303 10.1034/j.1600-0404.2003.00155.x

[B15] Ewing-Cobbs L Levin HS Fletcher JM Miner ME Eisenberg HM The children’s orientation and amnesia test: relationship to severity of acute head injury and to recovery of memory Neurosurgery 1990 27 683 2259396

[B16] Ponsford J Cameron P Fitzgerald M Grant M Mikocka-Walus A Long-term outcomes after uncomplicated mild traumatic brain injury: a comparison with trauma controls J Neurotrauma 2011 28 937 946 21410321 10.1089/neu.2010.1516

[B17] Yeates KO Taylor HG Neurobehavioural outcomes of mild head injury in children and adolescents Pediatr Rehabil 2005 8 5 16 15799131 10.1080/13638490400011199

[B18] McCrory P Meeuwisse W Johnston K Dvorak J Aubry M Molloy M Cantu R Consensus statement on concussion in sport: the 3rd international conference on concussion in sport held in Zurich, november 2008 Br J Sports Med 2009 43 Suppl 1 i76 i90 19433429 10.1136/bjsm.2009.058248

[B19] Cernak I Chang T Ahmed FA Cruz MI Vink R Stoica B Faden AI Pathophysiological response to experimental diffuse brain trauma differs as a function of developmental age Dev Neurosci 2010 32 442 453 20948187 10.1159/000320085

[B20] Creed JA DiLeonardi AM Fox DP Tessler AR Raghupathi R Concussive brain trauma in the mouse results in acute cognitive deficits and sustained impairment of axonal function J Neurotrauma 2011 28 547 563 21299360 10.1089/neu.2010.1729 PMC3070143

[B21] Giza CC Hovda DA The neurometabolic cascade of concussion J Athl Train 2001 36 228 235 12937489 PMC155411

[B22] Giza CC Maria NSS Hovda DA N-methyl-D-aspartate receptor subunit changes after traumatic injury to the developing brain J Neurotrauma 2006 23 950 961 16774479 10.1089/neu.2006.23.950 PMC2531140

[B23] Gosselin N Saluja RS Chen JK Bottari C Johnston K Ptito A Brain functions after sports-related concussion: insights from event-related potentials and functional MRI Phys Sportsmed 2010 38 27 37 20959693 10.3810/psm.2010.10.1805

[B24] Henninger N Sicard KM Li Z Kulkarni P Dutzmann S Urbanek C Schwab S Fisher M Differential recovery of behavioral status and brain function assessed with functional magnetic resonance imaging after mild traumatic brain injury in the rat Crit Care Med 2007 35 2607 2614 17828037 10.1097/01.CCM.0000286395.79654.8D

[B25] Henry LC Tremblay S Boulanger Y Ellemberg D Lassonde M Neurometabolic changes in the acute phase after sports concussions correlate with symptom severity J Neurotrauma 2010 27 65 76 19761385 10.1089/neu.2009.0962

[B26] Lipton ML Gellella E Lo C Gold T Ardekani BA Shifteh K Bello JA Branch CA Multifocal white matter ultrastructural abnormalities in mild traumatic brain injury with cognitive disability: a voxel-wise analysis of diffusion tensor imaging J Neurotrauma 2008 25 1335 1342 19061376 10.1089/neu.2008.0547

[B27] Shrey DW Griesbach GS Giza CC The pathophysiology of concussions in youth Phys Med Rehabil Clin N Am 2011 22 577 602 vii 22050937 10.1016/j.pmr.2011.08.002 PMC3211100

[B28] O’Connell KM Littleton-Kearney MT The role of free radicals in traumatic brain injury Biol Res Nurs 2013 15 253 263 22345426 10.1177/1099800411431823

[B29] Sanders MJ Sick TJ Perez-Pinzon MA Dietrich WD Green EJ Chronic failure in the maintenance of long-term potentiation following fluid percussion injury in the rat Brain Res 2000 861 69 76 10751566 10.1016/s0006-8993(00)01986-7

[B30] Sick TJ Pérez-Pinzón MA Feng ZZ Impaired expression of long-term potentiation in hippocampal slices 4 and 48 h following mild fluid-percussion brain injury in vivo Brain Res 1998 785 287 292 9518654 10.1016/s0006-8993(97)01418-2

[B31] Solomon GS Ott SD Lovell MR Long-term neurocognitive dysfunction in sports: what is the evidence? Clin Sports Med 2011 30 165 167 xi 21074090 10.1016/j.csm.2010.09.002

[B32] Vagnozzi R Signoretti S Cristofori L Alessandrini F Floris R Isgrò E Ria A Marziali S Zoccatelli G Tavazzi B Del Bolgia F Sorge R Broglio SP McIntosh TK Lazzarino G Assessment of metabolic brain damage and recovery following mild traumatic brain injury: a multicentre, proton magnetic resonance spectroscopic study in concussed patients Brain 2010 133 3232 3242 20736189 10.1093/brain/awq200

[B33] Len TK Neary JP Cerebrovascular pathophysiology following mild traumatic brain injury Clin Physiol Funct Imaging 2011 31 85 93 21078064 10.1111/j.1475-097X.2010.00990.x

[B34] Gurkanlar D Coven I Erdem R Ozen O Kosdak S Yilmaz C Yucel E Altinors N The effect of repetitious concussions on cognitive functions in rats Turk Neurosurg 2010 20 442 448 20963692 10.5137/1019-5149.JTN.3176-10.1

[B35] Nakajima Y Horiuchi Y Kamata H Yukawa M Kuwabara M Tsubokawa T Distinct time courses of secondary brain damage in the hippocampus following brain concussion and contusion in rats Tohoku J Exp Med 2010 221 229 235 20562525 10.1620/tjem.221.229

[B36] Green R Koshimori Y Turner G Research digest. Understanding the organic basis of persistent complaints in mTBI: findings from functional and structural neuroimaging Neuropsychol Rehabil 2010 20 471 478 20486011 10.1080/09602011003693298

[B37] Meehan WP III Medical therapies for concussion Clin Sports Med 2011 30 115 124 ix 21074086 10.1016/j.csm.2010.08.003 PMC3359788

[B38] Mittenberg W Burton DB A survey of treatments for post-concussion syndrome Brain Inj 1994 8 429 437 7951205 10.3109/02699059409150994

[B39] Al Sayegh A Sandford D Carson AJ Psychological approaches to treatment of postconcussion syndrome: a systematic review J Neurol Neurosurg Psychiatry 2010 81 1128 1134 20802219 10.1136/jnnp.2008.170092

[B40] Alsalaheen BA Mucha A Morris LO Whitney SL Furman JM Camiolo-Reddy CE Collins MW Lovell MR Sparto PJ Vestibular rehabilitation for dizziness and balance disorders after concussion J Neurol Phys Ther 2010 34 87 93 20588094 10.1097/NPT.0b013e3181dde568

[B41] Leddy JJ Kozlowski K Donnelly JP Pendergast DR Epstein LH Willer B A preliminary study of subsymptom threshold exercise training for refractory post-concussion syndrome Clin J Sport Med 2010 20 21 27 20051730 10.1097/JSM.0b013e3181c6c22c

[B42] Majerske CW Mihalik JP Ren D Collins MW Reddy CC Lovell MR Wagner AK Concussion in sports: postconcussive activity levels, symptoms, and neurocognitive performance J Athl Train 2008 43 265 274 18523563 10.4085/1062-6050-43.3.265 PMC2386420

[B43] Schneider KJ Iverson GL Emery CA McCrory P Herring SA Meeuwisse WH The effects of rest and treatment following sport-related concussion: a systematic review of the literature Br J Sports Med 2013 47 304 307 23479489 10.1136/bjsports-2013-092190

[B44] Thiagarajan P Ciuffreda KJ Capo-Aponte JE Ludlam DP Kapoor N Oculomotor neurorehabilitation for reading in mild traumatic brain injury (mTBI): an integrative approach NeuroRehabilitation 2014 34 129 146 24284470 10.3233/NRE-131025

[B45] McCrory P Collie A Anderson V Davis G Can we manage sport related concussion in children the same as in adults? Br J Sports Med 2004 38 516 519 15388528 10.1136/bjsm.2004.014811 PMC1724912

[B46] Wade DT Crawford S Wenden FJ King NS Moss NE Does routine follow up after head injury help? A randomised controlled trial J Neurol Neurosurg Psychiatry 1997 62 478 484 9153604 10.1136/jnnp.62.5.478 PMC486856

[B47] Ponsford J Willmott C Rothwell A Cameron P Kelly AM Nelms R Curran C Impact of early intervention on outcome following mild head injury in adults J Neurol Neurosurg Psychiatry 2002 73 330 332 12185174 10.1136/jnnp.73.3.330 PMC1738009

[B48] Wade DT King NS Wenden FJ Crawford S Caldwell FE Routine follow up after head injury: a second randomised controlled trial J Neurol Neurosurg Psychiatry 1998 65 177 183 9703167 10.1136/jnnp.65.2.177 PMC2170203

[B49] Beauchamp K Mutlak H Smith WR Shohami E Stahel PF Pharmacology of traumatic brain injury: where is the “golden bullet”? Mol Med 2008 14 731 740 18769636 10.2119/2008-00050.Beauchamp PMC2527342

[B50] Zafonte R Friedewald WT Lee SM Levin B Diaz-Arrastia R Ansel B Eisenberg H Timmons SD Temkin N Novack T Ricker J Merchant R Jallo J The citicoline brain injury treatment (COBRIT) trial: design and methods J Neurotrauma 2009 26 2207 2216 19803786 10.1089/neu.2009.1015 PMC2824223

[B51] Tenovuo O Pharmacological enhancement of cognitive and behavioral deficits after traumatic brain injury Curr Opin Neurol 2006 19 528 533 17102689 10.1097/WCO.0b013e328010944f

[B52] Gravel J D’Angelo A Carrière B Crevier L Beauchamp MH Chauny J-M Wassef M Chaillet N Interventions provided in the acute phase for mild traumatic brain injury: a systematic review Syst Rev 2013 2 63 23924958 10.1186/2046-4053-2-63 PMC3750385

[B53] Tal G Tirosh E Rehabilitation of children with traumatic brain injury: a critical review Pediatr Neurol 2013 48 424 431 23668865 10.1016/j.pediatrneurol.2012.11.008

[B54] Label LS Treatment of post-traumatic headaches: maprotiline or amitriptyline Neurology 1991 41 247

[B55] Watanabe TK Bell KR Walker WC Schomer K Systematic review of interventions for post-traumatic headache MR 2012 4 129 140 10.1016/j.pmrj.2011.06.003 22373462

[B56] Tyler GS McNeely HE Dick ML Treatment of post-traumatic headache with amitriptyline Headache 1980 20 213 216 7390803 10.1111/j.1526-4610.1980.hed2004213.x

[B57] Saran A Antidepressants not effective in headache associated with minor closed head injury Int J Psychiatry Med 1988 18 75 83 3294202 10.2190/15ah-jb7q-u94t-jeaf

[B58] Whyte J Hart T Schuster K Fleming M Polansky M Coslett HB Effects of methylphenidate on attentional function after traumatic brain injury. A randomized, placebo-controlled trial Am J Phys Med Rehabil 1997 76 440 450 9431261 10.1097/00002060-199711000-00002

[B59] Plenger PM Dixon CE Castillo RM Frankowski RF Yablon SA Levin HS Subacute methylphenidate treatment for moderate to moderately severe traumatic brain injury: a preliminary double-blind placebo-controlled study Arch Phys Med Rehabil 1996 77 536 540 8831468 10.1016/s0003-9993(96)90291-9

[B60] Green LB Hornyak JE Hurvitz EA Amantadine in pediatric patients with traumatic brain injury: a retrospective, case-controlled study Am J Phys Med Rehabil 2004 83 893 897 15624567 10.1097/01.phm.0000143400.15346.c8

[B61] Kuczynski A Crawford S Bodell L Dewey D Barlow KM Characteristics of post-traumatic headaches in children following mild traumatic brain injury and their response to treatment: a prospective cohort Dev Med Child Neuro 2013 55 636 641 10.1111/dmcn.12152 23560811

[B62] Acuña-Castroviejo D López LC Escames G López A García JA Reiter RJ Melatonin-mitochondria interplay in health and disease Curr Top Med Chem 2011 11 221 240 21244359 10.2174/156802611794863517

[B63] Camins A Sureda FX Junyent F Verdaguer E Folch J Beas-Zarate C Pallas M An overview of investigational antiapoptotic drugs with potential application for the treatment of neurodegenerative disorders Expert Opin Investig Drugs 2010 19 587 604 10.1517/13543781003781898 20402598

[B64] Esposito E Cuzzocrea S Antiinflammatory activity of melatonin in central nervous system Curr Neuropharmacol 2010 8 228 21358973 10.2174/157015910792246155 PMC3001216

[B65] Herrera F Sainz RM Mayo JC Martín V Antolín I Rodriguez C Glutamate induces oxidative stress not mediated by glutamate receptors or cystine transporters: protective effect of melatonin and other antioxidants J Pineal Res 2001 31 356 362 11703566 10.1034/j.1600-079x.2001.310411.x

[B66] Dai X Cui S Li S Chen Q Wang R Melatonin attenuates the development of antinociceptive tolerance to delta-, but not to mu-opioid receptor agonist in mice Behav Brain Res 2007 182 21 27 17568695 10.1016/j.bbr.2007.04.018

[B67] Dehghan F Khaksari Hadad M Asadikram G Najafipour H Shahrokhi N Effect of melatonin on intracranial pressure and brain edema following traumatic brain injury: role of oxidative stresses Arch Med Res 2013 44 251 258 23608674 10.1016/j.arcmed.2013.04.002

[B68] Redman J Armstrong S Ng KT Free-running activity rhythms in the rat: entrainment by melatonin Science 1983 219 1089 6823571 10.1126/science.6823571

[B69] Redman JR Armstrong SM Reentrainment of rat circadian activity rhythms: effects of melatonin J Pineal Res 1988 5 203 215 3367270 10.1111/j.1600-079x.1988.tb00782.x

[B70] Underwood H Goldman BD Vertebrate circadian and photoperiodic systems: role of the pineal gland and melatonin J Biol Rhythms 1987 2 279 315 2979667 10.1177/074873048700200404

[B71] Maldonado MD Murillo-Cabezas F Terron MP Flores LJ Tan DX Manchester LC Reiter RJ The potential of melatonin in reducing morbidity-mortality after craniocerebral trauma J Pineal Res 2007 42 1 11 17198533 10.1111/j.1600-079X.2006.00376.x

[B72] Carpentieri A Díaz de Barboza G Areco V Peralta López M TolosadeTalamoni N New perspectives in melatonin uses Pharmacol Res 2012 65 437 444 22311380 10.1016/j.phrs.2012.01.003

[B73] Leon J Acuña-Castroviejo D Sainz RM Mayo JC Tan DX Reiter RJ Melatonin and mitochondrial function Life Sci 2004 75 765 790 15183071 10.1016/j.lfs.2004.03.003

[B74] León J Acuña-Castroviejo D Escames G Tan D-X Reiter RJ Melatonin mitigates mitochondrial malfunction J Pineal Res 2005 38 1 9 15617531 10.1111/j.1600-079X.2004.00181.x

[B75] Allegra M Reiter RJ Tan D-X Gentile C Tesoriere L Livrea MA The chemistry of melatonin’s interaction with reactive species J Pineal Res 2003 34 1 10 12485365 10.1034/j.1600-079x.2003.02112.x

[B76] Rosen J Than NN Koch D Poeggeler B Laatsch H Hardeland R Interactions of melatonin and its metabolites with the ABTS cation radical: extension of the radical scavenger cascade and formation of a novel class of oxidation products, C2-substituted 3-indolinones J Pineal Res 2006 41 374 381 17014695 10.1111/j.1600-079X.2006.00379.x

[B77] Tan DX Manchester LC Reiter RJ Qi WB Karbownik M Calvo JR Significance of melatonin in antioxidative defense system: reactions and products Biol Signals Recept 2000 9 137 159 10899700 10.1159/000014635

[B78] Rodriguez C Mayo JC Sainz RM Antolín I Herrera F Martín V Reiter RJ Regulation of antioxidant enzymes: a significant role for melatonin J Pineal Res 2004 36 1 9 14675124 10.1046/j.1600-079x.2003.00092.x

[B79] Kotler M Rodríguez C Sáinz RM Antolín I Menéndez-Peláez A Melatonin increases gene expression for antioxidant enzymes in rat brain cortex J Pineal Res 1998 24 83 89 9510432 10.1111/j.1600-079x.1998.tb00371.x

[B80] Reiter RJ Paredes SD Korkmaz A Jou M-J Tan D-X Melatonin combats mo1lecular terrorism at the mitochondrial level Interdiscip Toxicol 2008 1 137 149 21218104 10.2478/v10102-010-0030-2 PMC2993480

[B81] Reiter RJ Tan D-X Fuentes-Broto L Melatonin: a multitasking molecule Prog Brain Res 2010 181 127 151 20478436 10.1016/S0079-6123(08)81008-4

[B82] Acuña-Castroviejo D Escames G León J Carazo A Khaldy H Mitochondrial regulation by melatonin and its metabolites Adv Exp Med Biol 2003 527 549 557 15206773 10.1007/978-1-4615-0135-0_63

[B83] Cardinali DP Pagano ES Scacchi Bernasconi PA Reynoso R Scacchi P Melatonin and mitochondrial dysfunction in the central nervous system Horm Behav 2013 63 322 330 22391273 10.1016/j.yhbeh.2012.02.020

[B84] Beni SM Kohen R Reiter RJ Tan DX Shohami E Melatonin-induced neuroprotection after closed head injury is associated with increased brain antioxidants and attenuated late-phase activation of NF-kappaB and AP-1 FASEB J 2004 18 149 151 14597558 10.1096/fj.03-0323fje

[B85] Koh JO Effect of snowboard-related concussion safety education for recognizing possible concussions J Sports Med Phys Fitness 2011 51 625 632 22212265

[B86] Tsai MC Chen WJ Tsai MS Ching CH Chuang JI Melatonin attenuates brain contusion-induced oxidative insult, inactivation of signal transducers and activators of transcription 1, and upregulation of suppressor of cytokine signaling-3 in rats J Pineal Res 2011 51 233 245 21545521 10.1111/j.1600-079X.2011.00885.x

[B87] Campolo M Ahmad A Crupi R Impellizzeri D Morabito R Esposito E Cuzzocrea S Combination therapy with melatonin and dexamethasone in a mouse model of traumatic brain injury J Endocrinol 2013 217 291 301 23532863 10.1530/JOE-13-0022

[B88] Das A Belagodu A Reiter RJ Ray SK Banik NL Cytoprotective effects of melatonin on C6 astroglial cells exposed to glutamate excitotoxicity and oxidative stress J Pineal Res 2008 45 117 124 18373557 10.1111/j.1600-079X.2008.00582.x PMC2632944

[B89] Paula-Lima AC Louzada PR De Mello FG Ferreira ST Neuroprotection against Abeta and glutamate toxicity by melatonin: are GABA receptors involved? Neurotox Res 2003 5 323 327 14715451 10.1007/BF03033152

[B90] Louzada PR Paula Lima AC Mendonca-Silva DL Noël F De Mello FG Ferreira ST Taurine prevents the neurotoxicity of beta-amyloid and glutamate receptor agonists: activation of GABA receptors and possible implications for Alzheimer’s disease and other neurological disorders FASEB J 2004 18 511 518 15003996 10.1096/fj.03-0739com

[B91] Andrews-Zwilling Y Bien-Ly N Xu Q Li G Bernardo A Yoon SY Zwilling D Yan TX Chen L Huang Y Apolipoprotein E4 causes age- and Tau-dependent impairment of GABAergic interneurons, leading to learning and memory deficits in mice J Neurosci 2010 30 13707 13717 20943911 10.1523/JNEUROSCI.4040-10.2010 PMC2988475

[B92] Maldonado MD Manfredi M Ribas-Serna J Garcia-Moreno H Calvo JR Melatonin administrated immediately before an intense exercise reverses oxidative stress, improves immunological defenses and lipid metabolism in football players Physiol Behav 2012 105 1099 1103 22212240 10.1016/j.physbeh.2011.12.015

[B93] Ochoa JJ Díaz-Castro J Kajarabille N García C Guisado IM De Teresa C Guisado R Melatonin supplementation ameliorates oxidative stress and inflammatory signaling induced by strenuous exercise in adult human males J Pineal Res 2011 51 373 380 21615492 10.1111/j.1600-079X.2011.00899.x

[B94] Velkov ZA Velkov YZ Galunska BT Paskalev DN Tadjer AV Melatonin: quantum-chemical and biochemical investigation of antioxidant activity Eur J Med Chem 2009 44 2834 2839 19168264 10.1016/j.ejmech.2008.12.017

[B95] Ozdemir D Tugyan K Uysal N Sonmez U Sonmez A Acikgoz O Ozdemir N Duman M Ozkan H Protective effect of melatonin against head trauma-induced hippocampal damage and spatial memory deficits in immature rats Neurosci Lett 2005 385 234 239 15970378 10.1016/j.neulet.2005.05.055

[B96] Ozdemir D Uysal N Gonenc S Acikgoz O Sonmez A Topcu A Ozdemir N Duman M Semin I Ozkan H Effect of melatonin on brain oxidative damage induced by traumatic brain injury in immature rats Physiol Res 2005 54 631 637 15720160

[B97] Tsai J Whealin JM Scott JC Harpaz-Rotem I Pietrzak RH Examining the relation between combat-related concussion, a novel 5-factor model of posttraumatic stress symptoms, and health-related quality of life in Iraq and Afghanistan veterans J Clin Psychiatry 2012 73 1110 1118 22781019 10.4088/JCP.11m07587

[B98] Wang WZ Fang X-H Stephenson LL Khiabani KT Zamboni WA Melatonin reduces ischemia/reperfusion-induced superoxide generation in arterial wall and cell death in skeletal muscle J Pineal Res 2006 41 255 260 16948786 10.1111/j.1600-079X.2006.00361.x

[B99] Borlongan CV Yamamoto M Takei N Kumazaki M Ungsuparkorn C Hida H Sanberg PR Nishino H Glial cell survival is enhanced during melatonin-induced neuroprotection against cerebral ischemia FASEB J 2000 14 1307 1317 10877823 10.1096/fj.14.10.1307

[B100] Görgülü A Palaoglu S Ismailoglu Ö Tuncel M Sürücü MT Erbil M Klnç K Effect of melatonin on cerebral edema in rats Neurosurgery 2001 49 1434 11846944 10.1097/00006123-200112000-00024

[B101] Samantaray S Sribnick EA Das A Knaryan VH Matzelle DD Yallapragada AV Reiter RJ Ray SK Banik NL Melatonin attenuates calpain upregulation, axonal damage and neuronal death in spinal cord injury in rats J Pineal Res 2008 44 348 357 18086148 10.1111/j.1600-079X.2007.00534.x PMC2613550

[B102] Samantaray S Das A Thakore NP Matzelle DD Reiter RJ Ray SK Banik NL Therapeutic potential of melatonin in traumatic central nervous system injury J Pineal Res 2009 47 134 142 19627458 10.1111/j.1600-079X.2009.00703.x PMC11877319

[B103] Manda K Anzai K Kumari S Bhatia AL Melatonin attenuates radiation-induced learning deficit and brain oxidative stress in mice Acta Neurobiol Exp (Wars) 2007 67 63 70 17474322 10.55782/ane-2007-1633

[B104] Rios ERV Venâncio ET Rocha NFM Woods DJ Vasconcelos S Macedo D Sousa FCF Fonteles MMF Melatonin: pharmacological aspects and clinical trends Int J Neurosci 2010 120 583 590 20707632 10.3109/00207454.2010.492921

[B105] Kumar A Singh A Possible involvement of GABAergic mechanism in protective effect of melatonin against sleep deprivation-induced behaviour modification and oxidative damage in mice Fundam Clin Pharmacol 2009 23 439 448 19709322 10.1111/j.1472-8206.2009.00737.x

[B106] Wilhelmsen M Amirian I Reiter RJ Rosenberg J Gögenur I Analgesic effects of melatonin: a review of current evidence from experimental and clinical studies J Pineal Res 2011 51 270 277 21615490 10.1111/j.1600-079X.2011.00895.x

[B107] Dhanaraj E Nemmani KVS Ramarao P Melatonin inhibits the development of tolerance to U-50,488H analgesia via benzodiazepine-GABAAergic mechanisms Pharmacol Biochem Behav 2004 79 733 737 15582681 10.1016/j.pbb.2004.10.002

[B108] Dodge NN Wilson GA Melatonin for treatment of sleep disorders in children with developmental disabilities J Child Neurol 2001 16 581 584 11510929 10.1177/088307380101600808

[B109] Peres MFP Zukerman E da Cunha Tanuri F Moreira FR Cipolla-Neto J Melatonin, 3 mg, is effective for migraine prevention Neurology 2004 63 757 15326268 10.1212/01.wnl.0000134653.35587.24

[B110] Miano S Parisi P Pelliccia A Luchetti A Paolino MC Villa MP Melatonin to prevent migraine or tension-type headache in children Neurol Sci 2008 29 285 287 18810607 10.1007/s10072-008-0983-5

[B111] Citera G Arias MA Maldonado-Cocco JA Lázaro MA Rosemffet MG Brusco LI Scheines EJ Cardinalli DP The effect of melatonin in patients with fibromyalgia: a pilot study Clin Rheumatol 2000 19 9 13 10752492 10.1007/s100670050003

[B112] Mozaffari S Rahimi R Abdollahi M Implications of melatonin therapy in irritable bowel syndrome: a systematic review Curr Pharm Des 2010 16 3646 3655 21128901 10.2174/138161210794079254

[B113] Yousaf F Seet E Venkatraghavan L Abrishami A Chung F Efficacy and safety of melatonin as an anxiolytic and analgesic in the perioperative period: a qualitative systematic review of randomized trials Anesthesiology 2010 113 968 976 20823763 10.1097/ALN.0b013e3181e7d626

[B114] van Geijlswijk IM van der Heijden KB Egberts ACG Korzilius HPLM Smits MG Dose finding of melatonin for chronic idiopathic childhood sleep onset insomnia: an RCT Psychopharmacology (Berl) 2010 212 379 391 20668840 10.1007/s00213-010-1962-0 PMC2952772

[B115] Song GH Leng PH Gwee KA Moochhala SM Ho KY Melatonin improves abdominal pain in irritable bowel syndrome patients who have sleep disturbances: a randomised, double blind, placebo controlled study Gut 2005 54 1402 1407 15914575 10.1136/gut.2004.062034 PMC1774717

[B116] Lu WZ Gwee KA Moochhalla S Ho KY Melatonin improves bowel symptoms in female patients with irritable bowel syndrome: a double-blind placebo-controlled study Aliment Pharmacol Ther 2005 22 927 934 16268966 10.1111/j.1365-2036.2005.02673.x

[B117] Saha L Malhotra S Rana S Bhasin D Pandhi P A preliminary study of melatonin in irritable bowel syndrome J Clin Gastroenterol 2007 41 29 32 17198061 10.1097/MCG.0b013e31802df84c

[B118] Alstadhaug KB Odeh F Salvesen R Bekkelund SI Prophylaxis of migraine with melatonin: a randomized controlled trial Neurology 2010 75 1527 1532 20975054 10.1212/WNL.0b013e3181f9618c

[B119] Acufla-Castroviejo D Escames G Macks M Hoyos AM Carballo AM Arauzo M Montes R Vives F Minireview: cell protective role of melatonin in the brain J Pineal Res 1995 19 57 63 8609597 10.1111/j.1600-079x.1995.tb00171.x

[B120] Antolín I Mayo JC Sainz RM del Brío MA Herrera F Martín V Rodríguez C Protective effect of melatonin in a chronic experimental model of Parkinson’s disease Brain Res 2002 943 163 173 12101038 10.1016/s0006-8993(02)02551-9

[B121] Kuczynski A Crawford S Bodell L Dewey D Barlow KM Characteristic of post-traumatic headaches after pediatric mild traumatic brain injury Can J Neur Sci 2011 38 no. s 10.1111/dmcn.12152 23560811

[B122] Antonaci F Sjaastad O Cervicogenic headache: a real headache Curr Neurol Neurosci Rep 2011 11 149 155 21125430 10.1007/s11910-010-0164-9

[B123] Obermann M Keidel M Diener H-C Post-traumatic headache: is it for real? Crossfire debates on headache: pro Headache 2010 50 710 715 20456158 10.1111/j.1526-4610.2010.01644.x

[B124] Donaldson CJ Hoffer ME Balough BJ Gottshall KR Prognostic assessments of medical therapy and vestibular testing in post-traumatic migraine-associated dizziness patients Otolaryngol Head Neck Surg 2010 143 820 825 21109084 10.1016/j.otohns.2010.09.024

[B125] Erickson JC Treatment outcomes of chronic post-traumatic headaches after mild head trauma in US soldiers: an observational study Headache 2011 51 932 944 21592097 10.1111/j.1526-4610.2011.01909.x

[B126] Bayly PV Black EE Pedersen RC Leister EP Genin GM In vivo imaging of rapid deformation and strain in an animal model of traumatic brain injury J Biomech 2006 39 1086 1095 16549098 10.1016/j.jbiomech.2005.02.014 PMC1479313

[B127] Wilhelmsen M Rosenberg J Gögenur I Anxiolytical, analgesic and sedative effects of melatonin in the perioperative phase Ugeskr Laeger 2011 173 1424 1427 Danish 21586247

[B128] Ayr LK Yeates KO Taylor HG Browne M Dimensions of postconcussive symptoms in children with mild traumatic brain injuries J Int Neuropsychol Soc 2009 15 19 30 19128525 10.1017/S1355617708090188 PMC2832119

[B129] Petersen C Scherwath A Fink J Koch U Health-related quality of life and psychosocial consequences after mild traumatic brain injury in children and adolescents Brain Inj 2008 22 215 221 18297593 10.1080/02699050801935245

[B130] Ganesalingam K Yeates KO Ginn MS Taylor HG Dietrich A Nuss K Wright M Family burden and parental distress following mild traumatic brain injury in children and its relationship to post-concussive symptoms J Pediatr Psychol 2008 33 621 629 18227110 10.1093/jpepsy/jsm133 PMC2839359

[B131] McCarthy ML MacKenzie EJ Durbin DR Aitken ME Jaffe KM Paidas CN Slomine BS Dorsch AM Berk RA Christensen JR Ding R CHAT Study Group The pediatric quality of life inventory: an evaluation of its reliability and validity for children with traumatic brain injury Arch Phys Med Rehabil 2005 86 1901 1909 16213229 10.1016/j.apmr.2005.03.026

[B132] Brooks BL Sherman EM Computerized neuropsychological testing to rapidly evaluate cognition in pediatric patients with neurologic disorders J Child Neurol 2012 27 8 982 991 22290863 10.1177/0883073811430863

[B133] Reynolds CR Kamphaus RW The clinician’s guide to the Behavior Assessment System for Children (BASC) 2002 New York: Guilford Press

[B134] Doyle A Ostrander R Skare S Crosby RD August GJ Convergent and Criterion-related Validity of the Behavior Assessment System for Children-Parent Rating Scale J Clin Child Psychol 1997 26 3 276 284 9292385 10.1207/s15374424jccp2603_6

[B135] Ayalon L Borodkin K Dishon L Kanety H Dagan Y Circadian rhythm sleep disorders following mild traumatic brain injury Neurology 2007 68 1136 1140 17404196 10.1212/01.wnl.0000258672.52836.30

[B136] Garcia J Rosen G Mahowald M Circadian rhythms and circadian rhythm disorders in children and adolescents Seminars in Pediatric Neurology 2001 8 4 229 240 11768785 10.1053/spen.2001.29044

[B137] Hofstra WA de Weerd AW How to assess circadian rhythm in humans: a review of literature Epilepsy Behav 2008 13 438 444 18588999 10.1016/j.yebeh.2008.06.002

[B138] Donders J DenBraber D Vos L Construct and criterion validity of the behaviour rating inventory of executive function (BRIEF) in children referred for neuropsychological assessment after pediatric traumatic brain injury J Neuropsychol 2010 4 197 209 19930791 10.1348/174866409X478970

[B139] Giza CC Kutcher JS Ashwal S Barth J Getchius TS Gioia GA Gronseth GS Guskiewicz K Mandel S Manley G McKeag DB Thurman DJ Zafonte R Summary of evidence-based guideline update: evaluation and management of concussion in sports: report of the guideline development subcommittee of the American academy of neurology Neurology 2013 80 2250 2257 23508730 10.1212/WNL.0b013e31828d57dd PMC3721093

[B140] Glass KL Natale MJ Janusz GA Gioia GA Anderson S Initial Development of a Parent Report of Post Concussion Symptoms in Children and Adolescents. Paper Presented at the Thirty-Third Annual Meeting of the International Neuropsychology Society: February 2-5 2005 2005 St Louis, MO: Cambridge Journals 171

[B141] Janusz JA Sady MS Gioia GA Postconcussion Symptom Assessment Mild Traumatic Brain Injury in Children and Adolescents, Volume 1 2012 New York: Guilford Press 241 263

[B142] Gioia GA Vaugh CG Isquith PK Manual for Pediatric Immediate Post-Concussion Assessment and Cognitive Testing 2011 Pittsburgh: IMPACT Applications

[B143] Jacobson NS Truax P Clinical significance: A statistical approach to defining meaningful change in psychotherapy research J Consult Clin Psycholog 1991 13 1 12 19 10.1037//0022-006x.59.1.12 2002127

[B144] Buscemi N Vandermeer B Hooton N Pandya R Tjosvold L Hartling L Baker G Klassen TP Vohra S The efficacy and safety of exogenous melatonin for primary sleep disorders. A meta-analysis J Gen Intern Med 2005 20 1151 1158 16423108 10.1111/j.1525-1497.2005.0243.x PMC1490287

[B145] Buscemi N Vandermeer B Hooton N Pandya R Tjosvold L Hartling L Vohra S Klassen TP Baker G Efficacy and safety of exogenous melatonin for secondary sleep disorders and sleep disorders accompanying sleep restriction: meta-analysis BMJ 2006 332 385 393 16473858 10.1136/bmj.38731.532766.F6 PMC1370968

