# Supplementary material for: A double-blind, placebo-controlled intervention trial of 3 and 10 mg sublingual melatonin for post-concussion syndrome in youths (PLAYGAME): study protocol for a randomized controlled trial
Source: Trials. 2014 Jul 7;15:271. doi: 10.1186/1745-6215-15-271 (PMC4227124; doi:10.1186/1745-6215-15-271)
Supplement: Additional file 1 — PLAYGAME trial metadata. [file 1745-6215-15-271-S1.docx]

| **Data Category** | **Information** |
| --- | --- |
| Primary registry and trial identifying number | ClinicalTrials.gov: NCT01874847 |
| Date of registration in primary registry | 6-Jun-13 |
| Secondary identifying numbers | Health Canada: 16397; CIHR 2933; CHREB 13-0372 |
| Source(s) of monetary or material support | Canadian Institutes of Health (CIHR) #2933; |
| Primary sponsor | CIHR |
| Contact for public queries | Principal Investigator: Karen Barlow karen.barlow@albertahealthservices.ca;  Brenda Turley Research Co-ordinator: 403 955 7814 |
| Contact for scientific queries | Karen Barlow karen.barlow@albertahealthservices.ca |
| Public Title | Play Game: Post-Concussion Syndrome in Youth |
| Scientific Title | Play Game: Post-Concussion Syndrome in Youth - a double-blinded, randomized placebo controlled trial of Melatonin |
| Countries of Recruitment | Canada |
| Health Condition | Post-concussion syndrome after mild traumatic brain injury |
| Intervention | Active comparator:*Melatonin* 10 mg or 3mg sublingual tablet per day for 28 days |
|  | Placebo comparator: Sugar pill lactose (matching sublingual tablet containing no active ingredients) |
| Key inclusion and exclusion criteria | *Inclusion Criteria:*   - Ages 13-19 years - Concussion/mild traumatic brain injury trauma with an increase in symptoms (increase in PCS symptoms compared with pre-injury status) at 30 days post injury   *Exclusion Criteria:*   - Previous significant medical history, or previous concussion within 12 months - Participant in a natural history study of concussion - Lactose intolerance, as the placebo contains lactose - Use of drugs that are likely to affect TMS, fMRI and/or sleep - Inability to complete questionnaires/evaluation e.g. non-English language - Claustrophobia/inability to tolerate MRI including ferromagnetic implants - Contraindications to TMS (including history of seizures, unexplained loss of consciousness (LOC), metal in the head and/or implanted brain medical devices, cardiac pacemaker, etc.) |
|  |  |
|  |  |
|  |  |
|  |  |
|  |  |
|  |  |
|  |  |
|  |  |
| Date of first enrolment | 20/1/2014 |
| Target sample size | 99 |
| Recruitment status | Recruiting |
| Primary outcome(s) | Change on the Post-Concussion Symptom Inventory questionnaire - Parent and Adolescent versions |
| Secondary outcome measures | - Change on the Child Health Questionnaire (CHQ) [Time Frame: Baseline, 4 and 12 weeks] - Change on the Behavior Assessment System for Children (BASC)-2 -Parent [Time Frame: Baseline, 4 and 12 weeks] - Behavior Rating Inventory of Executive Function (BRIEF) [Time Frame: Baseline, 4 and 12 weeks] - CNS Vital Signs [Time Frame: Baseline, 4 and 12 weeks] - Sleep parameters (Actigraphy) [Time Frame: Baseline, mid treatment and 4 weeks] |
|  |  |
|  |  |
|  |  |
|  |  |

Issue Date: 12 Nov 2013

Protocol Amendment Number: 01

Author(s): K.M.B; D.D.

Revision Chronology:

| 2013-June-6 | Original |
| --- | --- |
| 2013-Nov-12 | Amendment 01: Primary reason for amendment:  Changes regarding formulation of the comparator placebo from oral to sublingual preparation (matched for shape, size, taste and colour)  Changes regarding Melatonin 3mg and 10mg sublingual tablets encapsulation - encapsulation is no longer necessary.  Addition of first morning urine collection for measurement of Melatonin levels [Timing: pre-treatment, mid treatment and after treatment]. |
| 2014-Mar-24 | Amendment 02: Change to recruitment. Recruitment was changed to include local community sport medicine clinics and pediatric offices |
|  |  |
|  |  |
